# A study protocol for a randomized controlled trial of family-partnered delirium prevention, detection, and management in critically ill adults: the ACTIVATE study

**DOI:** 10.1186/s12913-020-05281-8

**Published:** 2020-05-24

**Authors:** Kirsten M. Fiest, Karla D. Krewulak, Bonnie G. Sept, Krista L. Spence, Judy E. Davidson, E. Wesley Ely, Andrea Soo, Henry T. Stelfox

**Affiliations:** 1 Department of Critical Care Medicine, University of Calgary & Alberta Health Services, Calgary, Canada; 2 Department of Community Health Sciences & O’Brien Institute of Public Health, University of Calgary, Calgary, Canada; 3 Department of Psychiatry & Hotchkiss Brain Institute, University of Calgary, Calgary, Canada; 4 Department of Psychiatry, UC San Diego School of Medicine, San Diego, California USA; 5 Tennessee Valley Veteran’s Affairs Geriatric Research Education Clinical Center (VA GRECC), Critical Illness, Brain Dysfunction, and Survivorship (CIBS) Center, Vanderbilt University Medical Center, Nashville, TN USA

**Keywords:** Delirium, Critical care, Family-centered care, Education, Prevention

## Abstract

**Background:**

Delirium is very common in critically ill patients admitted to the intensive care unit (ICU) and results in negative long-term outcomes. Family members are also at risk of long-term complications, including depression and anxiety. Family members are frequently at the bedside and want to be engaged; they know the patient best and may notice subtle changes prior to the care team. By engaging family members in delirium care, we may be able to improve both patient and family outcomes by identifying delirium sooner and capacitating family members in care.

**Methods:**

The primary aim of this study is to determine the effect of family-administered delirium prevention, detection, and management in critically ill patients on family member symptoms of depression and anxiety, compared to usual care. One-hundred and ninety-eight patient-family dyads will be recruited from four medical-surgical ICUs in Calgary, Canada. Dyads will be randomized 1:1 to the intervention or control group. The intervention consists of family-partnered delirium prevention, detection, and management, while the control group will receive usual care. Delirium, depression, and anxiety will be measured using validated tools, and participants will be followed for 1- and 3-months post-ICU discharge. All analyses will be intention-to-treat and adjusted for pre-identified covariates. Ethical approval has been granted by the University of Calgary Conjoint Health Research Ethics Board (REB19–1000) and the trial registered. The protocol adheres to the Standard Protocol Items: Recommendations for Interventional Trials (SPIRIT) checklist.

**Discussion:**

Critically ill patients are frequently unable to participate in their own care, and partnering with their family members is particularly important for improving experiences and outcomes of care for both patients and families.

**Trial registration:**

Registered September 23, 2019 on Clinicaltrials.gov NCT04099472.

## Background

Patients admitted to the Intensive Care Unit (ICU) are the sickest in the healthcare system; they have complex medical problems that require urgent treatment with life sustaining technologies [1]. Within this context, one of the largest challenges in the care of critically ill patients is the development of delirium. Delirium (an acute confusional state characterized by fluctuating course, attention deficits, and severe disorganization of behavior [2]) is present in over half of all critically ill patients admitted to ICUs [3–8]. Delirium is associated with negative outcomes in critically ill patients, including longer hospital stays [9], increased risk of long-term cognitive impairment, and death [10–12].

Family members of critically ill patients are also exposed to high levels of stress and are at risk of developing stress-related complications during and after the ICU stay, including sleep disturbances, anxiety, and depression [13]. Witnessing delirium is distressing for families, who watch their loved one's suffering both during and after a hospital stay [14]. Families are essential to the journey of critically ill patients and their participation in care may improve outcomes for both patients and families [15–18]. For example, there was a decrease in symptoms of both anxiety and depression in families who witnessed cardiopulmonary resuscitation [16] and those who received information on what to expect at the end of life [17]. Family involvement in preventing, detecting, and managing delirium has the potential to produce similar positive effects. The *Facilitated Sensemaking* framework [19–21] provides a theoretical basis for patient- and family-oriented studies in the ICU; engaging families in decision making and care provision may enhance their ability to cope with a new and challenging situation. Patients may also benefit from greater inclusion of family members in the ICU. Increased family presence does not increase infection rates [22, 23] or adverse events [23] and may decrease cardiocirculatory complications [24] and reduce patient falls [25]. Delirium is a common and serious complication of critical illness that affects both patients and their family members. ICU patients, family members, providers and decision-makers (ICU stakeholders) recognize the enormity of this issue and stakeholder groups have identified delirium detection and management as a top quality improvement opportunity [26–30].

Despite high prevalence, detrimental effects, and priority as a quality improvement initiative, delirium is often overlooked in the ICU or assumed to be a normal part of a patients’ clinical course [31]. The importance of brain dysfunction in the ICU is increasingly recognized and although formal delirium screening programs have been implemented worldwide, the majority of cases remain undetected [31]. Family members may be able to notice subtle changes in patients’ cognition and behavior before an unfamiliar clinical observer [32]. The prevention and management of delirium is also challenging; the only guideline recommended therapies for delirium are non-pharmacological and there are presently no known effective pharmacotherapies for delirium [33]. Family members may be engaged in these non-pharmacological delirium prevention and management therapies at the bedside through activities such as re-orientation, familiarization, and mobilization [33]. Critically ill patients are frequently unable to participate in their own care; by partnering with their family members (e.g., immediate family, relatives, friends), we may be able to improve experiences and outcomes of care for both patients and families [10, 34, 35]. We hypothesize that structured engagement of family members in the detection of delirium symptoms and prevention and management of delirium using non-pharmacological strategies will decrease the number of symptoms of anxiety and depression in family members of critically ill patients and may decrease the duration and severity of delirium in critically ill patients.

### Study aims

#### Primary aim

To determine the effect of family-administered delirium prevention, detection, and management in critically ill patients on family member symptoms of depression and anxiety, compared to usual care.

#### Secondary aims

1. To determine the effect of family-administered delirium prevention, detection, and management in critically ill patients on family member symptoms of psychological distress, compared to usual care.
2. To determine the effect of family-administered delirium prevention, detection, and management on the prevalence (ever/never delirium as indicated by an Intensive Care Unit Delirium Screening Checklist [ICDSC] score ≥ 4), duration (total days of an ICDSC score ≥ 4), and severity of delirium (as indicated by the ICDSC score, which ranges from 0 to 8) in critically ill patients, compared to usual care.
3. To determine the effect of family-administered delirium prevention, detection, and management on the diagnosis of the patient with delirium (ICDSC score ≥ 4), compared to usual care.
4. To determine the effect of family-administered delirium prevention, detection, and management on family member knowledge of delirium in critically ill patients, compared to usual care.
5. To determine the effect of family-administered delirium prevention, detection, and management on the burden of delirium (e.g., feelings of helplessness) experienced by family members of critically ill patients, compared to usual care.

### Patient involvement

Inclusiveness, support, mutual respect, and co-building are the core principles that will guide this work [36]. Patient and family member (herein referred to as patient advisors) involvement in the current project began during five meetings held between September 10, 2018 and September 5, 2019; they participated in priority setting initiatives and group discussions alongside other stakeholders (e.g., researchers, clinicians, decision makers). The research questions, protocol, and this paper were jointly developed with patient advisors (BGS) on our team. The patient advisors for this project have worked with our team consenting and recruiting participants for projects in the ICU and will continue to do so for this study. The intervention and questionnaire packages were piloted with patient advisors to determine appropriateness of language and the time commitment required for completion. Patient advisors will be included as authors on resulting publications (including the current paper, BGS) and be directly involved in dissemination through presentation of results to scientific and lay audiences. Our multidisciplinary team of patient advisors, researchers, clinicians, and knowledge users have demonstrated a track record of research success in co-leading national peer-reviewed grants (including the funding for the proposed study) and publishing with patient advisors [26, 37–39]. All patient advisors are compensated for their time.

## Methods

### Study design

We will conduct a multicenter, non-inferiority randomized controlled trial with participants randomly assigned to family-partnered delirium prevention, detection, and management or control (standard patient care). Patients and family members will be recruited from four medical-surgical ICUs located in four hospitals (Foothills Medical Centre [FMC], Peter Lougheed Centre [PLC], Rockyview General Hospital [RGH], South Health Campus [SHC] within a publicly funded healthcare system (Alberta, Canada). FMC (28-beds), PLC (18-beds) and RGH (10-beds) are teaching hospitals, while SHC (10-beds) is a non-teaching hospital. All four ICUs are within the same health region, have the same bed structure (single bed, private room), open visitation policies and standard of delirium care. The research team has access to eCritical MetaVision, a population-based, bedside clinical information system which captures in real time demographic, clinical, and outcome data for all ICU patients in Alberta, including routinely collected clinical measures of delirium and sedation [40]. Patient demographics (e.g., birthdate, sex) and clinical characteristics (e.g., admitting SOFA and APACHE-II scores, ICDSC and RASS assessments, sedation, etc.) will be collected.

### Eligibility criteria

Consecutive, eligible (Table 1) patients with at least one family member (defined here as family, relatives, or friends) present in the ICU will be approached by a patient advisor or research assistant to participate (Additional file 1). The patient and family member will be enrolled in the study as a dyad. Participants will be recruited at any point during their ICU admission, and participation will occur to a maximum of 5 days, or until ICU discharge or death. Anticipated study flow is presented in Table 2. If more than one family member is present, the study team will enroll the family member most familiar with the patient or the familiar expected to visit the most (and provide complete data).

**Table 1:** Study Inclusion and Exclusion Criteria

	Dyad	
	*Patient*	*Family*
Inclusion Criteria	• Age 18 years of age or above	• Age 18 years of age or above
	• Family member present	• Present
	• Richmond Agitation Sedation Scale score ≥ −3 (eligible for delirium assessment)	
Exclusion Criteria	• Unable to provide informed consent (patient or family member)	• Unable to provide informed consent (patient or family member)
	• Inability to communicate with the research staff (e.g., no hearing impairment and must be fluent in English)	• Inability to communicate with the research staff (e.g., no hearing impairment and must be fluent in English)
	• Anticipated to remain in ICU for less 24 h	• Anticipated to remain in ICU for less 24 h
	• Primary neurological injury (e.g., severe traumatic brain injury, subarachnoid hemorrhage)	
	• Glasgow Coma Scale score < 9

**Table 2:** Schedule of enrolment, interventions, and assessments

Activity / assessment	CRF (Yes/No)	Staff Member	-1 Prescreening / consent	0 Baseline / Randomization	T1 Immediately post-randomization	T2 Daily for Remainder of ICU stay	F1 Follow-up 1-month	F2 Follow-up 3-months
Prescreening consent	No	Nurse unaffiliated with study	X					
Screening log	No	Research Assistant	X					
Consent form	No	Research Assistant	X					
Inclusion/Exclusion form	Yes	Research Assistant	X					
Randomization	No	Biostatistician unaffiliated with study		X				
Patient demographics form	Yes	Research Assistant		X				
Family member demographics form	Yes	Research Assistant		X				
Educational video/booklet	No	Research Assistant			X			
Delirium prevention and management	Yes	Research Assistant				X		
Delirium detection (Sour Seven)	Yes	Research Assistant				X		
CIDKQ	Yes	Research Assistant		X		X	X	X
GAD-7	Yes	Research Assistant		X		X	X	X
PHQ-9	Yes	Research Assistant		X		X	X	X
K-10	Yes	Research Assistant		X		X	X	X
DEL-B	Yes	Research Assistant		X		X		
ICDSC	No	Research Assistant		X		X		
RASS	No	Research Assistant		X		X		
CCFNI	Yes	Research Assistant		X				
BCQ	Yes	Research Assistant		X				
CSS	Yes	Research Assistant		X				

*Abbreviations*: *BCQ* Barriers to Care Questionnaire in the ICU, *CIDKQ* Caregiver ICU Delirium Knowledge Questionnaire, *CCFNI* Critical Care Family Needs Inventory, *CSS* Caregiver Coping Strategies, *DEL-B* Delirium Burden, *GAD-7* Generalized Anxiety Disorder 7, *ICU* Intensive Care Unit, *K-10* Kessler Psychological Distress Scale, *PHQ-9* Patient Health Questionnaire 9, *RASS* Richmond Agitation Sedation Scale

### Randomization

Participants will be randomized using computer-generated randomization to either the intervention group or the control group (1:1). Randomization will be stratified by site and restricted using permuted random blocks within each strata. The random allocation sequence will be implemented using the REDCap study database by a biostatistician unaffiliated with the study. This biostatistician will generate the random allocation sequence to assign participants to either the intervention or control groups. No one directly involved in the study will have access to the allocation sequence and this feature will be accessed exclusively by the unaffiliated biostatistician. The pre-determined allocation sequence from the biostatistician will be employed centrally (FMC) to assign participants to the intervention or control group. Research assistants will receive this information from the REDCap database once a patient is consented.

### Blinding

Due to the nature of the intervention, participants, care providers, and study staff will not be blinded to the intervention. The individuals collecting data and conducting the data analysis will be blinded to the assignment of interventions.

### Study intervention

Participants in both the intervention and control groups will receive an informational pamphlet developed by our team on ICU delirium (standard of care); this will be presented to all patients and families upon admission. In addition to the pamphlet, the intervention is comprised of two components: (1) an education module for family members to prevent, detect, and manage ICU delirium; and (2) delirium detection using the Sour Seven [41], a validated family member-administered ICU delirium detection tool [42]. Family members are not engaged in the assessment of delirium in usual care. To ensure consistency between patients, the study team will use a standardized wording to introduce the study and to answer common questions.

1. Education: Our team developed and pilot tested the educational materials, both paper (i.e., booklet) and digital (i.e., https://youtu.be/I88-Lohht64) formats, which included a video, hypothetical case vignettes, and a delirium knowledge questionnaire. We created the educational video with input from patients, family members, and members of the ICU care team. In addition our team modified an existing Caregiver Delirium Knowledge Questionnaire [43] to the ICU context to develop the Caregiver ICU Delirium Knowledge Questionnaire (CIDKQ) [44]. We developed case vignettes of hypothetical ICU patients based on the four features that, when found in combination, indicate delirium is present: sudden onset or fluctuating course AND inattention AND either altered level of consciousness OR disorganized thinking. **The CIDKQ** (Cronbach alpha = 0.80) and case vignettes (Sour Seven Sensitivity: 90.0%, Specificity: 65.0%) were validated in a cross-sectional study of 80 family members [44]. The **educational materials were validated** in a **quasi-experimental pre-test post-test study to validate** the education materials. Preliminary results demonstrate improvement in family member delirium knowledge after receiving delirium education (as measured by the CIDKQ) and improvement in identification of delirium in hypothetical ICU patients (Sour Seven Sensitivity: 100.0%, Specificity: 75%). This education module will be presented to family members on the signs of delirium, risk factors, delirium detection, and delirium prevention and management using non-pharmacological strategies. The material will be provided as a 6-min video or as a booklet. Family members will practice identifying delirium with the Sour Seven, using previously validated case vignettes of hypothetical ICU patients [44]. The education module will be provided on day one of the study by a patient advisor or research assistant.
2. **Family-administered delirium prevention, detection, and management:** Our team completed a **systematic review** to identify existing family member-administered delirium detection tools [45], which included the Family Confusion Assessment Method (FAM-CAM) [46] and Sour Seven [41] **and an observational study** of 147 patient-family member dyads by our team evaluated the reliability and validity of the FAM-CAM [46] and Sour Seven [41] in the ICU [39, 42]. The Sour Seven had superior diagnostic accuracy [(Sour Seven Sensitivity: 72.9%, Specificity: 68.8%) (FAM-CAM- Sensitivity: 54.1%, Specificity: 76.8%)], with family members preferring the Sour Seven and thus, the Sour Seven will be employed in the current study [39].
3. For up to five study days, family members will be provided a daily checklist of non-pharmacological interventions to prevent and manage delirium, which include patient orientation (e.g., noting date, time, and location), mobility (e.g., family-assisted mobility), and environmental protocols (e.g., providing glasses or ear plugs). They will complete the Sour Seven once daily to detect delirium in the patient.

### Timing of intervention

**Fig. 1:**
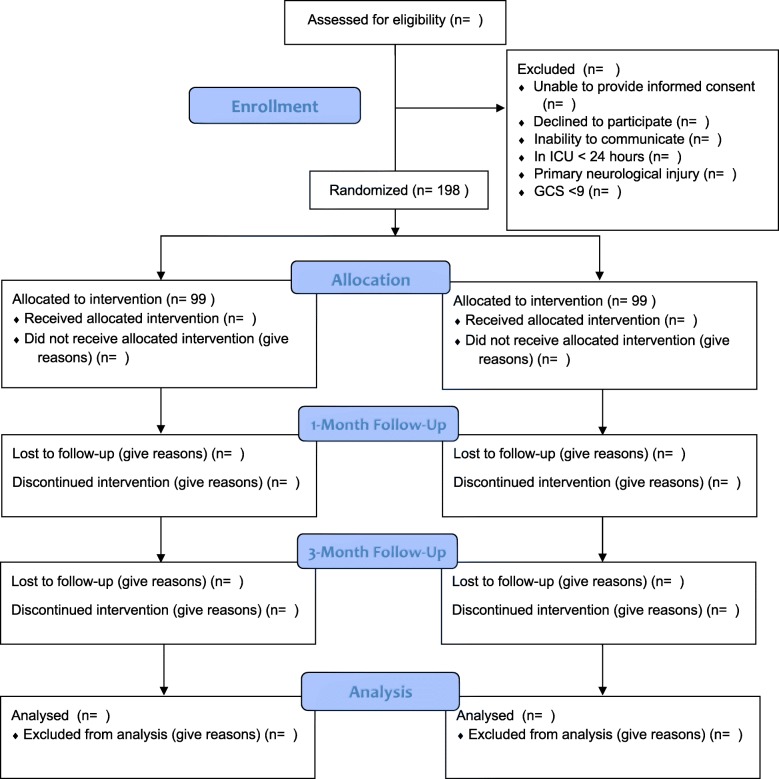
CONSORT Flow Diagram

The first day of intervention materials will be provided to the family upon enrollment (Fig. 1). The family will be provided ICU delirium education either by watching a 6-min video on their personal device or on a study tablet. If preferred, the family caregiver can receive the education by reading through a physical delirium education booklet with the research assistant or patient advisor (to ensure standardized provision of delirium education). Following the delirium education, the family will use the information contained in two case vignettes to practice completing the Sour Seven. Demographic questionnaires for the patient and family member will be administered on day one of the study. Knowledge of delirium on the CIDKQ [44] will be assessed prior to the intervention and immediately following administration of the education module. Delirium prevention and management strategies utilized by the family will be queried once daily (for 5 days maximum). The burden of delirium (Delirium Burden Scale [DEL-B]) on caregivers will be assessed daily for up to 5 days. Family delirium detection assessments (Sour Seven) will be conducted once daily over the course of the ICU stay (maximum 5 days). The results of the family delirium assessments will be shared with the bedside nurse by the research team. Family member psychiatric symptom assessments (Patient Health Questionnaire-9 [PHQ-9] and Generalized Anxiety Disorder-7 [GAD-7]) and distress (Kessler Psychological Distress Scale [K-10]) will be conducted daily over the course of the ICU stay (maximum of 5 days), with the distress and psychiatric questionnaires completed BEFORE the Sour Seven questionnaire is completed. Questionnaires on the caregiver’s ICU experience (Critical Care Family Needs Inventory [CCFNI]; Barriers to Care Questionnaire [BCQ]) and coping strategies (Coping Strategies Scale [CSS]), will be completed once over the course of the ICU stay (day 1).

#### Study follow-up

Follow-up of family members will occur at 1- and 3-months post-ICU discharge (Fig. 1). Our team has developed a secure, online survey site for questionnaire completion. For participants who prefer paper forms, a pre-addressed stamped envelope with questionnaires will be provided. We will send reminders to participants 1-week prior to the follow-up date, and continue to provide reminders up to 1-week following the follow-up date.

### Measurement of exposures

#### Caregiver ICU delirium knowledge questionnaire (CIDKQ)

The CIDKQ is a 21-item questionnaire to assess a caregiver’s delirium knowledge (e.g., risk factors, outcomes and symptoms). The questionnaire will be completed on day one of the study before and immediately after the education module, and at 1-month and 3-month follow-up post patient ICU discharge.

#### Sour Seven

The Sour Seven is a 7-item tool to detect delirium based on the Diagnostic and Statistical Manual of Mental Disorders (DSM) criteria [2] for delirium in patients, including those with dementia, and is designed to be administered by family members [41]. Assessed symptoms include reduced attention, an altered level of awareness, and disordered thinking. Scores of ≥9 out of 18 are indicative of delirium [41]. The questionnaire will be completed once daily for up to five study days.

#### Critical Care Family Needs Inventory (CCFNI)

The CCFNI is a 45-item tool that identifies caregiver needs across five domains: assurance, proximity, information, comfort, and support [47, 48].

#### Barriers to Care Questionnaire (BCQ)

The BCQ is a 39-item tool that assesses caregiver barriers regarding health knowledge and beliefs, expectations about care, skills, and marginalization [49]. Scores range from 0 to 100, with higher scores indicating fewer barriers [50].

#### Coping Strategies Scale (CSS)

The CSS is a 48-item questionnaire that assesses a caregiver’s coping strategies in response to stress caused by health-related issues [51].

#### Richmond Agitation and Sedation Scale (RASS)

The RASS is a 10-point agitation-sedation scale centered at 0 (indicates calm and alert), with scores ranging from + 4 to − 5; more negative scores indicate greater levels of sedation and more positive scores indicate higher levels of agitation [52]. The RASS score is completed by on-duty bedside nurses as part of standard care every 4 h.

### Measurement of outcomes

#### Family member outcomes

##### Generalized Anxiety Disorder-7 (GAD-7) (primary aim)

The GAD-7 is a validated 7-item scale following the DSM criteria for generalized anxiety disorder (GAD) over the previous 2 weeks [53]. Scores of ≥10 out of 21 are indicative of clinically significant GAD [53]. The questionnaire will be completed by caregivers (assessing their symptoms) once daily for up to five study days, and at 1-month and 3-month follow-up post patient ICU discharge.

##### Patient Health Questionnaire (PHQ-9) (primary aim)

The PHQ-9 is a validated 9-item scale for assessing symptoms of major depression in the past 2 weeks, based on DSM criteria for depression [54]. Scores of ≥10 out of 27 indicate clinically significant depression [54]. The questionnaire will be completed by caregivers (assessing their symptoms) once daily for up to five study days, and at 1-month and 3-month follow-up post patient ICU discharge.

##### Kessler Psychological Distress Scale (K-10) (secondary aim 1)

The K-10 is a 10-item tool to measure psychological distress and the likelihood of having a mental disorder [55] with higher scores indicating greater psychological distress. The questionnaire will be completed by caregivers (assessing their symptoms) once daily for up to five study days, and at 1-month and 3-month follow-up post patient ICU discharge.

##### Delirium Burden Scale (DEL-B) (secondary aim 5)

The DEL-B is an 8-item tool with two-level questions; the first level lists delirium burden features and if answered positive (yes), the follow-up question asks caregivers to rate how distressing the burden (e.g., feelings of helplessness, concern about increased responsibilities, not being recognized by patient) was on a 0–4 scale [56]. Total scores range between 0 and 40, with higher scores indicative of greater burden [56]. The DEL-B will be completed once daily for up to five study days.

#### Patient outcomes

##### Intensive Care Delirium Screening Checklist (ICDSC)

The ICDSC is an 8-item, delirium assessment tool for use in the ICU (1 point per item) [4]. An altered level of consciousness, along with the presence of three other symptoms, including inattention, psychomotor agitation/retardation, and disorientation are required to diagnose delirium. Scores of ≥4 out of 8 on the ICDSC are indicative of delirium (Sensitivity: 99%; Specificity: 64%) [4]. The ICDSC yields both a continuous measure of symptoms and a dichotomous diagnosis of delirium. The ICDSC is completed twice daily by on-duty bedside nurses as part of standard care.

### Statistical analysis plan

All data will be analyzed as intention to treat. Descriptive statistics will be used to summarize the patient and family characteristics. For all analyses, we will consider a two-tailed *p*-value < 0.05 to be statistically significant, and will adjust for multiple comparisons (Bonferroni correction) in the primary analysis where appropriate. For the secondary aims, we will not correct for multiple comparisons with statistical procedures, but rather consider them in our interpretation. Subgroup analyses and a priori *covariates* to be considered are: presence of mechanical ventilation, RASS score, delirium subtype, patient frailty status (based on Clinical Frailty Scale scores), patient age, patient sex, caregiver age, caregiver sex, and baseline levels of symptoms of depression, anxiety, and psychological distress. Linear regression models will be used to assess for effect modification and confounding. Missing data will be tabulated for all outcomes. If more than 5% of outcome data is missing, multiple imputation will be employed; for the primary aim, we will also conduct a sensitivity analysis assuming the data is missing not at random.

For the primary aim, we will report a difference in proportion of the clinically significant symptoms of depression [PHQ-9] and anxiety [GAD-7], mean PHQ-9 and GAD-7 scores, and frequency of categories of depression and anxiety (i.e., minimal, mild, moderate and severe) (at discharge from ICU, 1-, and 3- month follow-up) between control and intervention groups. For secondary aims we will report: 1) A difference in proportion of the most severe family members symptoms of psychological distress [K-10] (at discharge from ICU, 1-, and 3- month follow-up) between control and intervention groups. 2) Prevalence of delirium: Difference in proportion of patients with clinical delirium (ICDSC score ≥ 4) pre-post intervention (in-ICU) between control and intervention groups. Duration of delirium: Difference in mean days with clinical delirium (ICDSC score ≥ 4) pre-post intervention between control and intervention groups (in-ICU). Duration of delirium-free days: Difference in mean days without delirium on the ICDSC pre-post intervention between control and intervention groups (in-ICU). Severity of delirium: Difference in most severe ICDSC scores pre-post intervention between control and intervention groups (in-ICU). 3) Difference in the diagnosis of delirium in the medical chart, compared to usual care (when appropriate, i.e., when delirium is present) pre-post intervention between control and intervention groups (in-ICU). 4) Difference in mean CIDKQ scores pre-post intervention between control and intervention groups (in-ICU). 5) Difference in mean DEL-B scores pre-post intervention between control and intervention groups (in-hospital). The statistical significance of the differences between proportions or means (between the intervention and control groups) will be calculated using the.

#### Sample size calculation

*Primary Outcome:* To find a difference in the mean score on the PHQ-9 or GAD-7 of 3-points (from 13 to 10), with a standard deviation of 5-points, power of 90%, an alpha level of 0.05, and 20% loss to follow-up (based off of previous mortality rates in the study ICUs [local data]), 72 dyads per group are required (*N* = 144). *Secondary Outcomes:* To find a difference in the proportion of delirium events reported in the medical chart (most conservative estimate based on secondary outcomes selected) of 20% (from 10 to 30%) at 90% power, an alpha level of 0.05, and 20% loss to follow up, 99 dyads per group are required (*N* = 198). The primary outcome requires a smaller sample size, and thus the most conservative number based on a secondary outcome is employed here.

### Dissemination

Given the study carries minimal risk, a data safety and monitoring board has not been established. Team members (including patient advisors and trainees) will present the results of this work to a variety of knowledge users, including researchers and clinicians at research rounds and the public at open engagement sessions (e.g., Café Scientifique [37]) and patient-identified forums of interest. Traditional dissemination strategies will include the publication of results in a peer-reviewed journal and educational sessions, while we will employ non-traditional dissemination strategies including blog and social media posts targeted at a public audience. The study design adhered to the Standard Protocol Items: Recommendations for Interventional Trials (SPIRIT) checklist [57]. Any modifications to the protocol will be agreed upon by all protocol authors and approved by the ethics board prior to implementation.

### Registration

This study is registered on clinicaltrials.gov (NCT04099472).

### Study and data management

The coordinating centre for the study is the FMC in Calgary, Canada. Local investigators at each site will be responsible, along with a study coordinator, for day-to-day operations. Data will be managed in a REDCAP database created for this project and stored on a secure institutional network drive. Hard copy materials will be stored in a secure locked office in a locked cabinet. All data will only be accessible by study personnel.

## Discussion

Previous research demonstrates that family members of critically ill patients want to assist with nonpharmacological delirium prevention activities, but most family members do not possess enough delirium knowledge to be effective partners [58, 59]. A study by Black et al. reported family participation in psychological care does not improve the incidence of delirium [60]. These data will help us understand the effect that a family member’s participation in delirium prevention, detection and management will have on family-centered psychological outcomes.

The primary limitation for the ACTIVATE study is loss to follow-up after ICU discharge, which would threaten the internal validity of our findings through selection bias, should those who remain differ from those who are lost. Our experience recruiting in this population will help mitigate this issue; specifically the inclusion of patient advisors as recruiters, which we have found increases response rates dramatically (from 23% [39] to 75% [42]). Questionnaire completion will depend on the timing and availability of family members, thus missing data may also present a threat to our study through selection bias. We will ensure flexible opportunities for questionnaire completion to facilitate data capture and employ methods to impute missing data where appropriate. The burden of completing the questionnaires presents an additional limitation to the study; we have piloted the questionnaires with patient researchers on our team to ensure the length is manageable (average time less than 20 min).

## Supplementary information

**Additional file 1.** Study consent forms for patients and family members. Approved informed consent forms for both patients and family members.

**Additional file 2.** SPIRIT 2013 Checklist: Recommended items to address in a clinical trial protocol and related documents. SPIRIT checklist and corresponding page numbers in the manuscript.

## Data Availability

The study protocol is registered on clinicaltrials.gov (NCT04099472) and data and statistical code will be shared given reasonable requests to the corresponding author.

## Abbreviations

APACHE: Acute Physiology and Chronic Health Evaluation
BCQ: Barriers to Care Questionnaire
CCFNI: Critical Care Family Needs Inventory
CIDKQ: Caregiver ICU Delirium Knowledge Questionnaire
CONSORT: Consolidated Standards of Reporting Trials
CSS: Coping Strategies Scale
DEL-B: Delirium Burden Scale
DSM: Diagnostic and Statistical Manual of Mental Disorders
FAM-CAM: Family Confusion Assessment Method
FMC: Foothills Medical Centre
GAD: Generalized Anxiety Disorder
GAD-7: Generalized Anxiety Disorder-7 Item
ICDSC: Intensive Care Delirium Screening Checklist
ICU: Intensive Care Unit
K-10: Kessler-10
NCT: National Clinical Trial
PHQ-9: Patient Health Questionnaire-9 Item
PLC: Peter Lougheed Centre
RASS: Richmond Agitation and Sedation Scale
REB: Research Ethics Board
RGH: Rockyview General Hospital
SHC: South Health Campus
SOFA: Sequential Organ Failure Assessment
SPIRIT: Standard Protocol Items: Recommendations for Interventional Trials
