# Supplementary material for: A study protocol for a randomized controlled trial of family-partnered delirium prevention, detection, and management in critically ill adults: the ACTIVATE study
Source: BMC Health Serv Res. 2020 May 24;20:453. doi: 10.1186/s12913-020-05281-8 (PMC7245836; doi:10.1186/s12913-020-05281-8)
Supplement: Supplementary file 1 — Additional file 1. Study consent forms for patients and family members. Approved informed consent forms for both patients and family members. [file 12913_2020_5281_MOESM1_ESM.docx]

Additional File 1. Study consent forms for patients and family members

**Family-administered delirium prevention, detection, and management in the critically ill consent form**

TITLE: Family-administered delirium prevention, detection, and management in the critically ill

SPONSOR: University of Calgary and the Department of Critical Care Medicine

# INVESTIGATORS:

# Dr. Kirsten Fiest, PhD

Researcher, Assistant Professor, Department of Critical Care Medicine

Cumming School of Medicine, University of Calgary

Phone: 403-944-1478

Email: delirium@ucalgary.ca

This consent form is only part of the process of informed consent. It should give you the basic idea of what the research is about and what your participation will involve. If you would like more detail about something that is mentioned here, or information not included here, please ask. Take the time to read this carefully and to understand any accompanying information. You will receive a copy of this form.

**BACKGROUND**

It is common for patients who are treated in intensive care units (ICUs) to develop delirium. Nearly 50% of patients in Calgary intensive care units experience delirium during their ICU stay. A patient with delirium has a sudden change in thinking. A patient with delirium cannot pay attention, may see or hear things that are not there, be sleepier than usual, or agitated. Patients who have delirium are more likely to have longer ICU or hospital stays. They may also experience trouble with memory and attention once they leave the ICU. Delirium is also difficult for family members of patients who have delirium during and after their ICU stay. The detection of delirium in ICUs is difficult because many cases of delirium are missed. Specifically, delirium cases with “quieter” symptoms, like a patient who is sleepy.

Family caregivers are an underused resource for the identification, prevention and management of delirium. Family caregivers (family members, friends, formal caregivers) at the bedside know their family member the best. They are able to notice small changes in their thinking. Family caregivers may be very important partners for recognizing delirium symptoms. Many caregivers may have differing levels of knowledge on how to recognize, prevent, and manage delirium. This study will educate family caregivers on delirium symptoms, how to identify delirium using a multiple-choice questionnaire, and how to manage and prevent delirium using orientation, mobility, and environmental activities.

**WHAT IS THE PURPOSE OF THE STUDY?**

This study will measure if providing delirium prevention, detection, and management strategies to family members of critically ill patients improves:

1. Symptoms of psychological distress, depression and anxiety in family members.
2. The burden of delirium (based on a delirium burden questionnaire) on family members.
3. Delirium knowledge (based on a delirium knowledge questionnaire) in family members.
4. The frequency, duration, and/or severity of delirium in patients.
5. Documentation of patient delirium in the medical chart.

## WHAT WOULD I HAVE TO DO?

You are being asked to take part in this study because you are a patient admitted to the ICU and your family member is present. Your participation in this study will first involve the study team randomly choosing which group you will be part of.

- **Group 1**: Your involvement will include one step:

1. Fill out a single page demographic form (age, biological sex, gender, ethnicity, and education) once (2 minutes).

**OR**

- **Group 2**: Your involvement will include two steps:

1. Fill out a single page demographic form (age, biological sex, gender, ethnicity, relationship to patient and education) once (2 minutes).
2. Attempt the delirium prevention and management activities contained in the booklet, with help of your family caregiver. There are three parts. The first is an orientation protocol. It includes activities like providing visual and hearing aids, orientation of day/time/location, bringing in familiar objects from home, television during the day with daily news, and listening to non-verbal music. The second is a mobility protocol. The mobility protocol has brain games which may include passing a beach ball, working with play dough, sudoku, etc. These activities will depend on the patient’s ability. Last is an environmental protocol. Items in this section include turning lights off at night and on during the day, using ear plugs, reducing noise during the night.

If you would like to see copies of the demographic form or delirium prevention and management activities before deciding whether or not to participate in the study, please let a study team member know.

## WHAT ARE THE RISKS?

There are no expected risks associated with participating in this study.

**WILL I BENEFIT IF I TAKE PART?**

There may not be any direct benefits to you from this study. A study report will be written to inform local and provincial practices and policies about the education of family caregivers in the ICU. This report may inform future guidelines to support family caregiver and healthcare worker partnerships in delirium prevention, detection, and management.

## DO I HAVE TO PARTICIPATE?

Your participation in this study is voluntary. If you choose to participate, you may withdraw from the study at any time without jeopardizing your health care. You can withdraw from the study by notifying any member of the research study team. If you chose to withdraw from the study, you can ask that any of your data collected up to the point of withdrawal is excluded. Please note that it is not possible to withdraw data once they have been published or otherwise disseminated. You may also ask questions or ask for more information whenever you like.

**WILL I BE PAID FOR PARTICIPATING, OR DO I HAVE TO PAY FOR ANYTHING?**

You will not be paid for participating nor will you have to pay for anything.

**WILL MY RECORDS BE KEPT PRIVATE?**

If you and your family member consent to join the study, qualified members of the study team will have permission to review your medical charts to see how often delirium was recorded in the chart and if there were any other factors that may have impacted the results of the study (e.g., age, sex, illness severity). All information obtained during the study will be kept private and will only be accessed by the study team. Authorized representatives from the University of Calgary and the Conjoint Health Research Ethics Board may look at your identifiable medical/clinical study records for quality assurance purposes. All information will be stored in a secured area (i.e. locked filing cabinet and/or password protected computer). All information obtained during the study will be held in strict confidence. Confidentiality will be respected and no information that discloses your own or your family member’s identity will be released or published. Your decision on whether or not to take part in the study will not affect you or your family member’s future relations with the University of Calgary or Alberta Health Services.

## SIGNATURES

Your signature on this form indicates that you have understood to your satisfaction the information regarding your participation in the research project and agree to participate as a participant. In no way does this waive your legal rights nor release the investigators or involved institutions from their legal and professional responsibilities. You are free to withdraw from the study at any time without jeopardizing your health care. If you have further questions concerning matters related to this research, please contact:

Dr. Kirsten Fiest (403-944-1478)

If you have any questions concerning your rights as a possible participant in this research, please contact the Chair, Conjoint Health Research Ethics Board, University of Calgary at 403-220-7990.

| Participant’s Name |  | Signature and Date |
| --- | --- | --- |
|  |  |  |
| Investigator/Delegate’s Name |  | Signature and Date |
|  |  |  |
| Witness’ Name |  | Signature and Date |
|  |  |  |

Time of consent:

The University of Calgary Conjoint Health Research Ethics Board has approved this research study.

A signed copy of this consent form has been given to you to keep for your records and reference.

**Family-administered delirium prevention, detection, and management in the critically ill consent form**

TITLE: Family-administered delirium prevention, detection, and management in the critically ill

SPONSOR: University of Calgary and the Department of Critical Care Medicine

# INVESTIGATORS:

# Dr. Kirsten Fiest, PhD

Researcher, Assistant Professor, Department of Critical Care Medicine

Cumming School of Medicine, University of Calgary

Phone: 403-944-1478

Email: delirium@ucalgary.ca

This consent form is only part of the process of informed consent. It should give you the basic idea of what the research is about and what your participation will involve. If you would like more detail about something that is mentioned here, or information not included here, please ask. Take the time to read this carefully and to understand any accompanying information. You will receive a copy of this form.

**BACKGROUND**

It is common for patients who are treated in intensive care units (ICUs) to develop delirium. Nearly 50% of patients in Calgary intensive care units experience delirium during their ICU stay. A patient with delirium has a sudden change in thinking. A patient with delirium cannot pay attention, may see or hear things that are not there, be sleepier than usual, or agitated. Patients who have delirium are more likely to have longer ICU or hospital stays. They may also experience trouble with memory and attention once they leave the ICU. Delirium is also difficult for family members of patients who have delirium during and after their ICU stay. The detection of delirium in ICUs is difficult because many cases of delirium are missed. Specifically, delirium cases with “quieter” symptoms, like a patient who is sleepy.

Family caregivers are underused for the identification, prevention and management of delirium. Family caregivers (family members, friends, formal caregivers) at the bedside know their family member the best. They are able to notice small changes in their family member’s thinking. Family caregivers may be very important partners for recognizing delirium symptoms. Many caregivers may have differing levels of knowledge on how to recognize, prevent, and manage delirium. This study will educate family caregivers on delirium symptoms, how to identify delirium, and how to manage and prevent delirium.

**WHAT IS THE PURPOSE OF THE STUDY?**

This study will measure if providing delirium prevention, detection, and management strategies to family members of critically ill patients improves:

1. Symptoms of psychological distress, depression and anxiety in family members.
2. The burden of delirium (based on a delirium burden questionnaire) on family members.
3. Delirium knowledge (based on a delirium knowledge questionnaire) in family members.
4. The frequency, duration, and/or severity of delirium in patients.
5. Documentation of patient delirium in the medical chart.

## WHAT WOULD I HAVE TO DO?

You are being asked to take part in this study because you are a family caregiver of a patient admitted to the ICU. Your participation in this study will first involve the study team randomly choosing which group you will be part of.

- **Group 1**: Your involvement will include three steps:
- Complete the following questionnaires once after you enroll in the study. You will have the help of a research assistant (total time: 20-25 minutes)
  1. A single page demographic form (age, biological sex, gender, ethnicity, relationship to patient and education).
  2. Questionnaires to understand your ICU experience as a family caregiver. These are the Critical Care Family Needs Inventory (CCFNI) and the Barriers to Care for the Intensive Care Unit (BCQ-ICU). The CCFNI is a 45-item questionnaire. It measures the importance of certain needs in the ICU. The BCQ-ICU is a 39-item questionnaire. It measures the importance of certain problems caregivers may face in the ICU.
  3. A questionnaire for caregivers to report coping strategies that they use while caring for their loved one in the ICU. This is called the Caregiver Coping Strategies (CSS) questionnaire. The CSS is a 32-item multiple-choice questionnaire.
  4. A questionnaire to measure your baseline knowledge of delirium. This is the Caregiver Intensive Care Unit Delirium Knowledge Questionnaire (CIDKQ). The CIDKQ has 21 multiple-choice questions.
- Complete the following questionnaires daily, for a maximum of five (5) days. You will have the help of a research assistant (total time: 10-15 minutes/day):
  1. Questionnaires to understand the mental wellbeing of caregivers. These are the Kessler Psychological Distress Scale (KPDS-10), The Generalized Anxiety Disorder 7-item (GAD-7), and the Patient Health Questionnaire 9-item (PHQ-9). The KPDS-10 has 10 questions. The GAD-7 has 7 questions. The PHQ-9 has 9 questions. All ask about your mental health.
  2. A questionnaire to understand the burden that delirium can have on ICU caregivers. This is the Delirium Burden Questionnaire (DEL-B). The DEL-B is an 8-item multiple choice questionnaire that measures the distress of seeing features of delirium in your loved one.
- One month after leaving the ICU, you will receive an email link to complete an online survey. The questionnaires in this survey are the psychological outcome questionnaires (KPDS-10, GAD-7, PHQ-9) and the delirium knowledge questionnaire (CIDKQ). This will take about 10-15 minutes. Three months after leaving the ICU, you will receive an email link to complete another online survey. The questionnaires in this survey are the psychological outcome questionnaires (KPDS-10, GAD-7, PHQ-9) and the delirium knowledge questionnaire (CIDKQ). This will take about 10-15 minutes.

**OR**

- **Group 2**: Your involvement will include six steps:

1. Complete the following questionnaires once after you enroll in the study. You will have the help of a research assistant (total time: 25-30 minutes)
   1. A single page demographic form (age, biological sex, gender, ethnicity, relationship to patient and education).
   2. Questionnaires to understand your ICU experience as a family caregiver. These are the Critical Care Family Needs Inventory (CCFNI) and the Barriers to Care for the Intensive Care Unit (BCQ-ICU). The CCFNI is a 45-item questionnaire. It measures the importance of certain needs in the ICU. The BCQ-ICU is a 39-item questionnaire. It measures the importance of certain problems caregivers may face in the ICU.
   3. A questionnaire for caregivers to report coping strategies that they use while caring for their loved one in the ICU. This is the Caregiver Coping Strategies (CSS) questionnaire. The CSS is a 32-item multiple-choice questionnaire.
   4. A questionnaire to measure your baseline knowledge of delirium. This is the Caregiver Intensive Care Unit Delirium Knowledge Questionnaire (CIDKQ). The CIDKQ has 21 multiple-choice questions.
2. Complete the following questionnaires daily, for a maximum of five (5) days. You will have the help of a research assistant (total time: 15-20 minutes/day):
   1. Questionnaires to understand the mental wellbeing of caregivers. These are the Kessler Psychological Distress Scale (KPDS-10), The Generalized Anxiety Disorder 7-item (GAD-7), and the Patient Health Questionnaire 9-item (PHQ-9). The KPDS-10 has 10 questions. The GAD-7 has 7 questions. The PHQ-9 has 9 questions. All ask about your mental health.
   2. A questionnaire to understand the burden that delirium can have on ICU caregivers. This is the Delirium Burden Questionnaire (DEL-B). The DEL-B is an 8-item multiple choice questionnaire that measures the distress of seeing features of delirium in your loved one.
3. Watch a 6-minute educational video about ICU delirium. The video includes information on what delirium is and how to identify, prevent and manage it. You will also receive a booklet that includes practice assessing delirium using descriptions of hypothetical patients. This booklet is yours to keep. You will complete the CIDKQ right after to see if your delirium knowledge changed after watching the video and reading the booklet. This part will take about 15-20 minutes.
4. Assess your family member for delirium every day using the provided Sour Seven questionnaire for a maximum of five (5) days. The “Sour Seven” is a 7-item yes/no questionnaire. It asks about changes to your family member’s thinking and concentration. This questionnaire takes about 3 minutes.
5. Attempt the delirium prevention and management activities contained in the booklet. There are three parts. The first is an orientation protocol. It includes activities like providing visual and hearing aids, orientation of day/time/location, bringing in familiar objects from home, television during the day with daily news, and listening to non-verbal music. The second is a mobility protocol. The mobility protocol has brain games which may include passing a beach ball, working with play dough, sudoku, etc. These activities will depend on the patient’s ability. Last is an environmental protocol. Items in this section include turning lights off at night and on during the day, using ear plugs, reducing noise during the night.
6. One month after leaving the ICU, you will receive an email link to complete an online survey. The questionnaires in this survey are the psychological outcome questionnaires (KPDS-10, GAD-7, PHQ-9) and the delirium knowledge questionnaire (CIDKQ). This will take about 10-15 minutes. Three months after leaving the ICU, you will receive an email link to complete another online survey. The questionnaires in this survey are the psychological outcome questionnaires (KPDS-10, GAD-7, PHQ-9) and the delirium knowledge questionnaire (CIDKQ). This will take about 10-15 minutes.

If you would like to see copies of any of these questionnaires before deciding whether or not to take part in the study, please let a study team member know.

## WHAT ARE THE RISKS?

There are no expected risks associated with participating in this study.

**WILL I BENEFIT IF I TAKE PART?**

There may not be any direct benefits to you from this study. A study report will be written to inform local and provincial practices and policies about the education of family caregivers in the ICU. This report may inform future guidelines to support family caregiver and healthcare worker partnerships in delirium prevention, detection, and management.

## DO I HAVE TO PARTICIPATE?

Your participation in this study is voluntary. If you choose to participate, you may withdraw from the study at any time without jeopardizing your family member’s health care. You can withdraw from the study by notifying any member of the research study team. There may also be some days where you choose not to complete the questionnaires. Not completing the questionnaires from one day does not disqualify you from completing the rest of the study. If you chose to withdraw from the study, you can also ask that any of your data collected is excluded. Please note that it is not possible to withdraw data once they have been published or otherwise disseminated. You may also ask questions or ask for more information whenever you like.

**WILL I BE PAID FOR PARTICIPATING, OR DO I HAVE TO PAY FOR ANYTHING?**

You will not be paid for participating nor will you have to pay for anything.

**WILL MY RECORDS BE KEPT PRIVATE?**

If you and your family member consent to join the study, qualified members of the study team will have permission to review your family member’s medical charts. They will do this to see how often delirium was recorded in the chart, and if there were any other factors that may have impacted the results of the study (e.g., age, sex, illness severity). All information obtained during the study will be kept private and will only be accessed by the study team. Authorized representatives from the University of Calgary and the Conjoint Health Research Ethics Board may look at your family member’s identifiable medical/clinical study records for quality assurance purposes. All information will be stored in a secured area (i.e. locked filing cabinet and/or password protected computer). All information obtained during the study will be held in strict confidence. Confidentiality will be respected and no information that discloses your own or your family member’s identity will be released or published. Your decision on whether or not to take part in the study will not affect you or your family member’s future relations with the University of Calgary, or Alberta Health Services.

**WOULD YOU BE WILLING TO BE CONTACTED FOR PARTICIPATION IN FOCUS GROUPS TO SHARE FEEDBACK ON YOUR ICU EXPERIENCE?**

You are being invited to give consent for Dr. Kirsten Fiest or a qualified member of her study to contact you at some time in the future to invite you to participate in a further study. This study will explore your experience of caring for a critically ill patient. Please include your contact information below:

☐ [Specify, e.g., Telephone]:

☐ [Specify, e.g., E-mail]:

## SIGNATURES

Your signature on this form indicates that you have understood to your satisfaction the information regarding your participation in the research project and agree to participate as a participant. In no way does this waive your legal rights nor release the investigators or involved institutions from their legal and professional responsibilities. You are free to withdraw from the study at any time without jeopardizing your family member’s health care. If you have further questions concerning matters related to this research, please contact:

Dr. Kirsten Fiest (403-944-1478)

If you have any questions concerning your rights as a possible participant in this research, please contact the Chair, Conjoint Health Research Ethics Board, University of Calgary at 403-220-7990.

| Participant’s Name |  | Signature and Date |
| --- | --- | --- |
|  |  |  |
| Investigator/Delegate’s Name |  | Signature and Date |
|  |  |  |
| Witness’ Name |  | Signature and Date |
|  |  |  |

Time of consent:

The University of Calgary Conjoint Health Research Ethics Board has approved this research study.

A signed copy of this consent form has been given to you to keep for your records and reference.
